# Chondromyxoid fibroma of zygomatic bone: A case report

**DOI:** 10.1016/j.amsu.2022.103394

**Published:** 2022-02-22

**Authors:** Zainab Elzouiti, Achraf Sbai, Amal Bennani, Fahd Elayoubi, Adil Eabdenbi Tsen

**Affiliations:** a Department of Oral and Maxillofacial Surgery, Mohammed VI University Hospital, Faculty of Medicine and Pharmacy, Oujda, Morocco; b Department of ENT and Cervicofacial Surgery, Mohammed VI University Hospital, Faculty of Medicine and Pharmacy, Oujda, Morocco; c Department of Pathology, Mohammed VI University Hospital, Faculty of Medicine and Pharmacy, Oujda, Morocco

**Keywords:** Chondromyxoid fibroma, Zygoma, Conservative approach, Case report

## Abstract

**Introduction:**

Chondromyxoid fibroma (CMF) is a rare benign bone tumor that typically affects long bones, only 2% of CMFs involved facial bones or skull, zygomatic localization is extremely rare with only 8 cases reported in literature so far.

**Presentation of case:**

We report a case of 88 old years patient with painful swelling in the right zygomatic around 1 year, progressively increasing in volume, Computed tomography (CT) scan showed an osteolytic lesion in the right zygomatic bone with cortical destruction. Surgical management consisted of bone curettage using intra oral approach, the histopahological findings were in favor of the diagnosis of CMF.

**Discussion:**

CF is a rare bone tumor and represents less than 1% of all bone tumors, the maxillofacial bones are rarely affected, with the mandible as a site of predilection, the zygomatic location is extremely rare. The clinical presentation is not typical, radiologically, the lesion is usually osteolytic with well defined margins.En bloc resection is the gold standard, some authors recommand conservative approach to avoid esthetic and functional sequels.

**Conclusion:**

We reported a very rare presentation of CF involving zygomatic bone treated by conservative approach.

## Highlights

Chondromyxoid fibroma involving an extragnathic bone in facial skeleton is very rare
Clinical and radiological presentations are not specific
The classic histological finding of chondromyxoid fibroma is stellate or spindle-shaped cells arranged in lobules with a myxoid background
The surgical managment consisted of en bloc resection or conservative curettage

## 1 Introduction

Chondromyxoid fibroma (CMF) is a rare benign bone tumor of cartilaginous origin, accounting for less than 0.4–1% of all bone tumors. Long bones are the most affected, but occurrence in facial bones or the skull is rare with only 2% of cases [1].

CMF is generally misdiagnosed due to clinical and radiological non specificity [2].

En bloc resection is the gold standard, however some authors recommand conservative curettage with regular follow-up [3].

In this case we report a rare presentation of chondromyxoid fibroma involving the zygomatic bone, to our knowledge only 8 cases of zygomatic CMF have been reported in the literature [[1], [2], [3], [4], [5], [6], [7], [8]].

This case report has been reported in line with the SCARE Criteria [10].

## 2 Presentation of case

A 88 years old patient was admitted to our department of oral and maxillofacial surgery with a painful swelling in the right zygomatic region around 1 year, progressively increasing in volume, there was no clear history of personal or family history of any chronic disease, clinical examination finds a painful, fixe and firm mass of the right zygomatic bone, without skin abnormalities, intraoral examination finds an edentulous patient with a good mouth opening, without intraoral expression of the tumor.

The general blood test was normal.

**Fig. 1:**
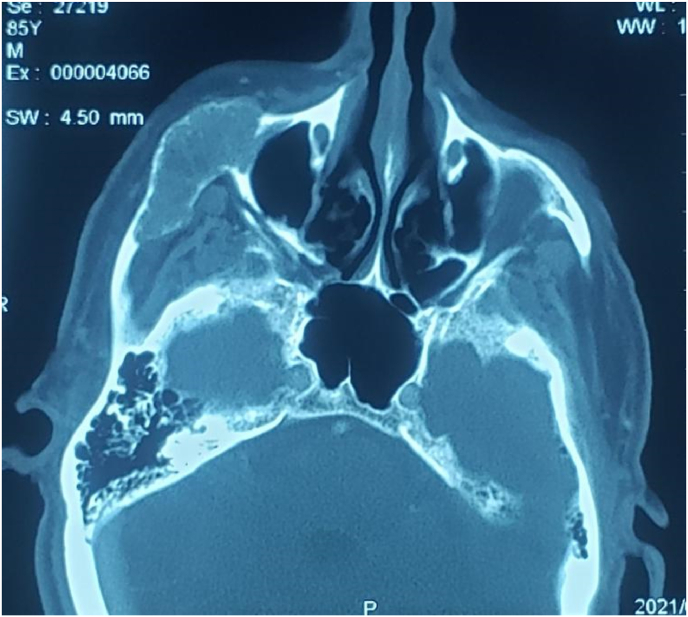
Computed tomography scan (axial view) revealed an osteolytic lesion in the right zygomatic bone.

Computed tomography (CT) scan revealed an osteolytic lesion involving the right zygomatic bone with cortical destruction, extended to the zygomatic arch (Fig. 1).

**Fig. 2:**
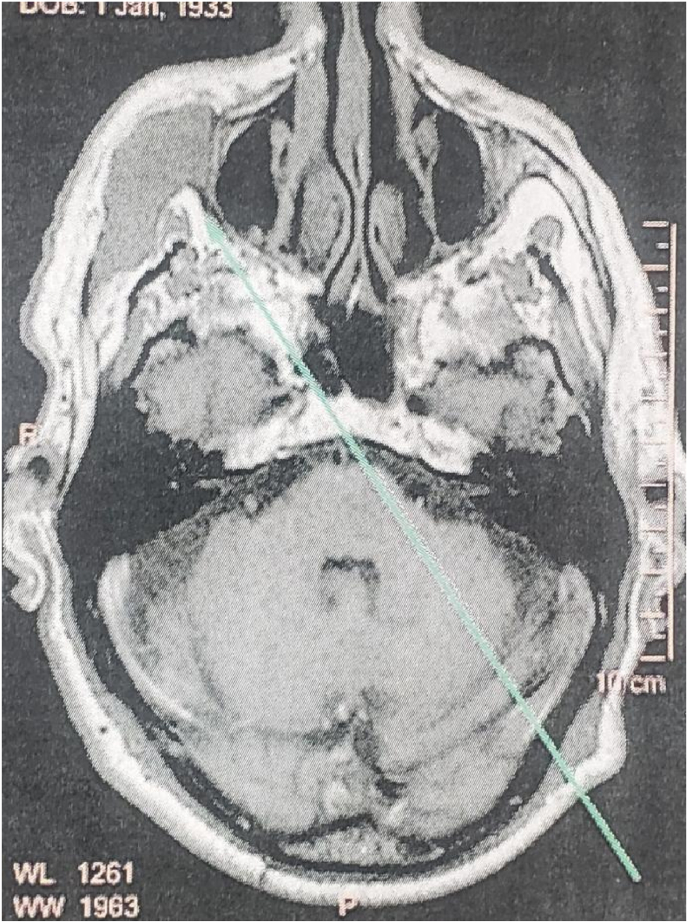
axial T1 sequence showed an hypointense zygomatic lesion with regular borders and lysis of the cortical bone.

Facial MRI with and without contrast was indicated and showed hypointense lobulated lesion of right zygoma on T1 sequence (Fig. 2), and hyperintense signal on T2 sequence.

**Fig. 3:**
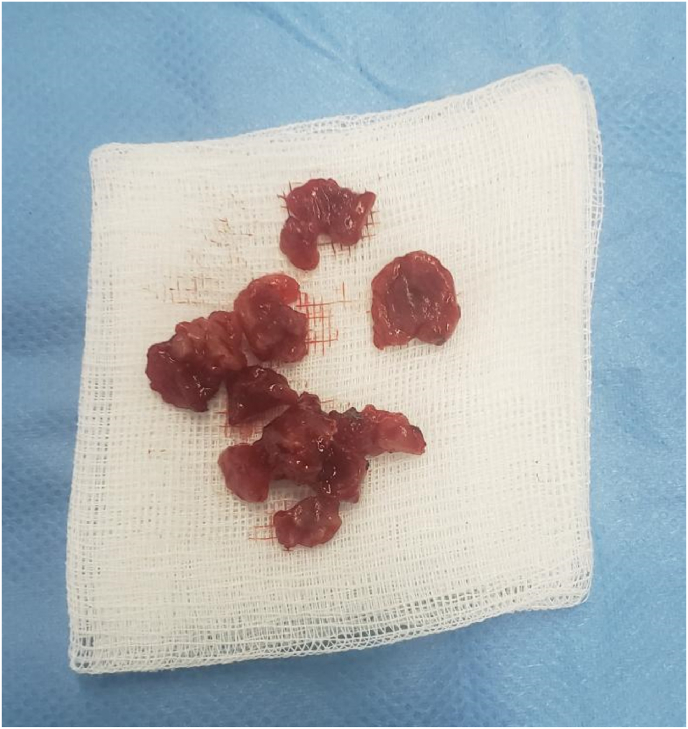
Surgical specimen after resection.

The surgical management was performed by a qualified professor with the aid of medical residents, it consisted of bone curettage by intra oral approach under general anaesthesia, the lesion was approached through an upper right vestibular incision(Fig. 3).

**Fig. 4:**
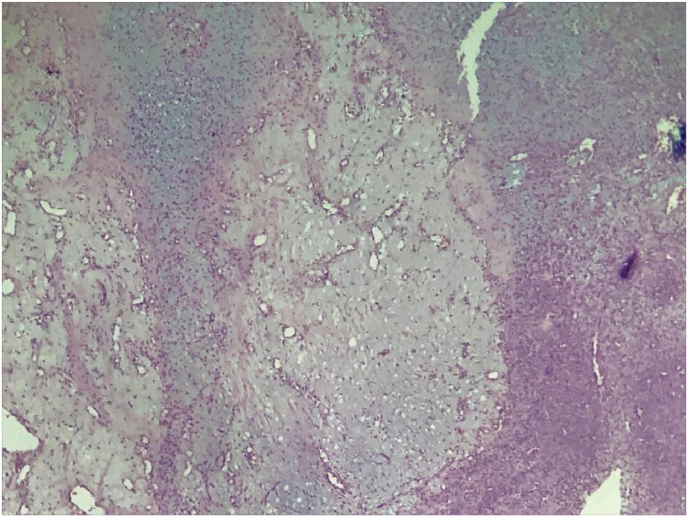
Microphotography showing a lobulated architecture tumor proliferation (HE, x 40).

**Fig. 5:**
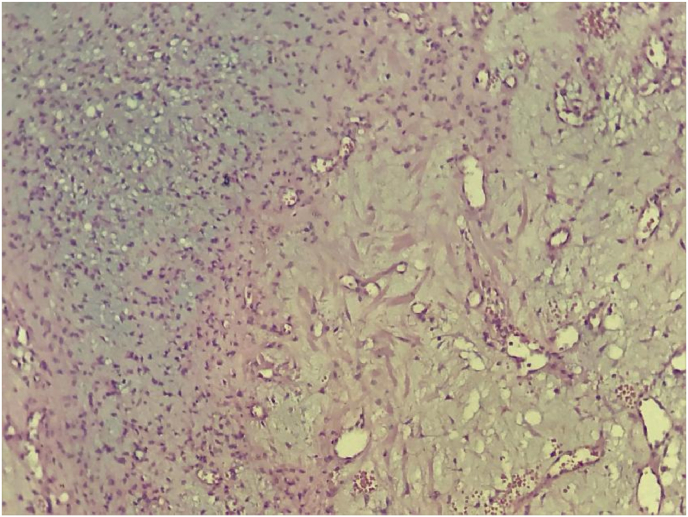
The lobules are delimited by small cells of chondroblastic appearance and centered by spindle-shaped or stellate cells (HE, x400).

The histopahological examination of the resected specimen showed multiples lobules (Fig. 4), with areas of hypercellularity at the center made up with stellate cells, which disposed in a myxoid background, at the periphery, the lobules are hypercellular with a spindle-shaped cells (Fig. 5), these findings were in favor of the diagnosis of CMF.

The post operative period was uneventful and the patient was discharged from the hospital at the 4th post operative day.

The patient is under regular follow-up with no sign of recurrence to date (5th post-operative month).

## 3 Discussion

CF is a rare bone tumor and represents less than 1% of all bone tumors, the tibia and distal femur are the most frequent locations, the maxillofacial bones are rarely affected, with the mandible as a site of predilection [1], the zygomatic location is extremely rare with only eight cases reported in litterature to our knowledge [[1], [2], [3], [4], [5], [6], [7], [8]].

The clinical presentation is not specific, it is characterized by an insidious pain or a progressively growing swelling, occasionally the tumor can be asymptomatic and fortuitously discovered on radiological exams, our case was admitted for local painful swelling.

Radiologic findings of CMF are not characteristic, the tumor usually appears as an osteolytic lobulated lesion with well defined margins which confirm the benign nature of the tumor, partial or complete cortical erosion is generally seen. Intralesional calcifications are punctiform and rarely found [2].

Only one case in the literature provide the MRI characterization of zygomatic CMF, the same findings are identified in our case [2].

MRI signal is generally heterogeneous. The lesion, with a non-specific signal, appears in iso- or hypointense T1, in hypersignal T2 and is enhanced heterogeneously after injection. Calcifications and bone trabeculations are hypointense on all sequences. Soft tissue extension is not frequent but possible.

Histologically, this tumor is defined by the World Health Organization (WHO) as “*a benign tumor characterized by lobules of spindle-shaped or stellate cells with abundant myxoid or chondroid intercellular material separated by zones of more cellular tissue rich in spindle-shaped or round cells with varying number of multinucleated giant cells of different sizes*” [9].

CMF is characterized by its lobulated architecture, there are variable presentations, a proportion of myxoid, chondroid and fibrous tissue is arranged in a lobular pattern made with stellate or spindle-shaped cells distributed in a myxoid background.

The lobules tend to be hypercellular in the periphery and hypocellular in the center, and are separated by abundant blood vessels, only few tumors show calcifications or mineralization.

The differential diagnosis includes chondrosarcoma and chondroblastoma, CMF must be distinguished from this tumors especially from chondrosarcoma because the management of this aggressive tumor is totally different and the resection must be large including free bone rims.

In chondrosarcoma the lobules are irregular, calcifications and malignant hyaline cartilage are easly identified. The distinction between chondroblastoma and CMF is not always easy, chondroblastoma is characterized by the presence of chondroblast-like cells and osteoclast-like giant cells with areas of focal calcification [9].

The surgical managment varied from conservative curettage to en bloc resection which constitutes the gold standard, conservative treatment with regular follow-up is recommended by many authors to avoid esthetic sequel of total tumor resection, especially when the lesion is localized in the facial skeleton, however recurrence rate of 25% has been reported after conservative approach [3].

## 4 Conclusion

CMF involving an extragnathic bone in facial skeleton is very rare, this tumor is generally misdiagnosed due to clinical and radiological non specificity, the diagnosis of certitude is histologic, the surgical resection is the gold standard, conservative treatment with regular monitoring is recommended by many authors to avoid esthetic sequel of total tumor resection, especially when the lesion is located in the facial skeleton.

## Provenance and peer review

Not commissioned, externally peer-reviewed.

## Sources of funding

We have any financial sources.

## Ethical approval

Not required.

## Consent

Written informed consent was obtained from the patient for publication of this case report and accompanying images. A copy of the written consent is available for review by the Editor-in-Chief of this journal on request.

## Author contribution

Dr. Zainab Elzouiti and Dr. Achraf Sbai wrote the manuscript and analysed the literature research, Pr. Adil Eabdenbi tsen, Pr. Fahd Elayoubi supervised the writing of manuscript and performed the scientific validation. Pr Amal Bennani provided the histopathological analysis. All authors read and approved the manuscript.

## Ethical approval

Not required.

## Please state any sources of funding for your research

We have any financial sources.

## Author contribution

Dr. Zainab Elzouiti and Dr. Achraf Sbai wrote the manuscript and analysed the literature research, Pr. Adil Eabdenbi tsen, Pr. Fahd Elayoubi supervised the writing of manuscript and performed the scientific validation. Pr Amal Bennani provided the histopathological analysis. All authors read and approved the manuscript.

## Please state any conflicts of interest

The authors have no conflicts of interest to declare.

## Registration of research studies

Name of the registry:
Unique Identifying number or registration ID
Hyperlink to your specific registration (must be publicly accessible and will be checked):

## Guarantor

Dr zainab elzouiti.

## Declaration of competing interest

The authors have no conflicts of interest to declare.
